# PD58 Health-Related Quality Of Life In Patients With Cutaneous Leishmaniasis In Brazil Using EQ-5D

**DOI:** 10.1017/S0266462325101906

**Published:** 2025-12-29

**Authors:** Janaína Carvalho, Sarah Nascimento Silva, Tália Machado de Assis, Endi Lanza Galvão, Mayra Moreira, Mônica Andrade, Kenya Noronha, Gláucia Cota

## Abstract

**Introduction:**

Cutaneous leishmaniasis (CL) is a neglected infectious disease with a known negative impact on health-related quality of life (HRQoL). However, no studies were found that quantitatively defined the health utility of CL. Our aim was to evaluate the impact of CL on HRQoL in patients attending a Brazilian reference center using the EQ-5D-3L visual analogue scale questionnaire to generate utility measures for CL.

**Methods:**

This cross-sectional study included patients with CL from a referral center for the disease in Minas Gerais, Brazil between 2020 and 2023. Patients were interviewed during active disease and retrospectively before the onset of disease symptoms. Each patient’s health status was assessed using the EQ-5D-3L questionnaire. The losses in HRQoL were estimated by comparing the proportion of responses before CL onset with those during its active phase. Socioeconomic data were collected using a standardized questionnaire, and sociodemographic and clinical data were collected directly from the medical records. This study was approved by the Fiocruz Minas ethics committee.

**Results:**

A total of 143 patients with a mean age of 52 years (standard deviation 17) were included, 73 percent of whom were male. The mean utility score before the onset of CL symptoms was 0.858. A comparison of the responses related to health status before and after the illness revealed significant losses (p<0.05) in all dimensions of the EQ-5D, especially those related to pain, malaise, and usual activities, which were reduced to 50 percent and 27 percent, respectively. CL also affected the median visual analogue scale score and utility scores, which were 87 and 70 and 0.858 and 0.716, respectively.

**Conclusions:**

These results confirmed the negative impact of CL on all dimensions assessed by the EQ-5D-3L visual analogue scale questionnaire, revealing another mechanism perpetuating the cycle of suffering and poverty surrounding neglected tropical diseases. These findings are essential for conducting future cost-utility studies, enabling comparisons of different treatments for this disease.

